# Identification and functional characterization of arginine vasopressin receptor 1A : atypical chemokine receptor 3 heteromers in vascular smooth muscle

**DOI:** 10.1098/rsob.170207

**Published:** 2018-01-31

**Authors:** Lauren J. Albee, Heather M. LaPorte, Xianlong Gao, Jonathan M. Eby, You-Hong Cheng, Amanda M. Nevins, Brian F. Volkman, Vadim Gaponenko, Matthias Majetschak

**Affiliations:** 1 Burn and Shock Trauma Research Institute, Department of Surgery, Loyola University Chicago Stritch School of Medicine, 2160 S. 1st Avenue, Maywood, IL 60153, USA; 2 Department of Molecular Pharmacology and Therapeutics, Loyola University Chicago Stritch School of Medicine, 2160 S. 1st Avenue, Maywood, IL 60153, USA; 3 Department of Biochemistry, Medical College of Wisconsin, Milwaukee, WI 53226, USA; 4 Department of Biochemistry and Molecular Genetics, University of Illinois at Chicago, Chicago, IL 60607, USA

**Keywords:** vasoconstriction, vasopressor, arginine vasopressin, CXCL11, CXCL12, ubiquitin

## Abstract

Recent observations suggest that atypical chemokine receptor (ACKR)3 and chemokine (C-X-C motif) receptor (CXCR)4 regulate human vascular smooth muscle function through hetero-oligomerization with α_1_-adrenoceptors. Here, we show that ACKR3 also regulates arginine vasopressin receptor (AVPR)1A. We observed that ACKR3 agonists inhibit arginine vasopressin (aVP)-induced inositol trisphosphate (IP_3_) production in human vascular smooth muscle cells (hVSMCs) and antagonize aVP-mediated constriction of isolated arteries. Proximity ligation assays, co-immunoprecipitation and bioluminescence resonance energy transfer experiments suggested that recombinant and endogenous ACKR3 and AVPR1A interact on the cell surface. Interference with ACKR3 : AVPR1A heteromerization using siRNA and peptide analogues of transmembrane domains of ACKR3 abolished aVP-induced IP_3_ production. aVP stimulation resulted in β-arrestin 2 recruitment to AVPR1A and ACKR3. While ACKR3 activation failed to cross-recruit β-arrestin 2 to AVPR1A, the presence of ACKR3 reduced the efficacy of aVP-induced β-arrestin 2 recruitment to AVPR1A. AVPR1A and ACKR3 co-internalized upon agonist stimulation in hVSMC. These data suggest that AVPR1A : ACKR3 heteromers are constitutively expressed in hVSMC, provide insights into molecular events at the heteromeric receptor complex, and offer a mechanistic basis for interactions between the innate immune and vasoactive neurohormonal systems. Our findings suggest that ACKR3 is a regulator of vascular smooth muscle function and a possible drug target in diseases associated with impaired vascular reactivity.

## 1. Background

The 7-transmembrane domain (TM) receptors chemokine (C-X-C motif) receptor 4 (CXCR4) and atypical chemokine receptor 3 (ACKR3) are involved in the regulation of vascular function and blood pressure control [1–7]. The underlying mechanisms, however, are not well understood. While CXCR4 is a typical G protein-coupled receptor (GPCR) that couples to Gα_i_ and recruits β-arrestin, ACKR3 does not couple to G proteins but recruits β-arrestin, leading to receptor internalization and G protein-independent signalling events [8,9]. Both receptors share C-X-C chemokine ligand 12 (CXCL12, stromal cell-derived factor 1) as a cognate agonist [8]. Furthermore, CXCR4 and ACKR3 are thought to form heteromeric complexes, resulting in preferential activation of β-arrestin signalling over canonical G protein signalling pathways upon activation with CXCL12 [10,11]. Besides heteromerization with ACKR3, CXCR4 may form heteromeric complexes with multiple other GPCRs, such as chemokine (C-C motif) receptor (CCR)2, CCR5, CXCR3, chemerin receptor 23, β_2_-adrenergic receptor (AR), δ-opioid receptor or cannabinoid receptor 2, leading to altered pharmacological properties of the interacting receptor partners [11–18].

Recently, we reported cross-talk between CXCR4, ACKR3 and α_1_-ARs in vascular smooth muscle, through which activation of the chemokine receptors regulates α_1_-AR-mediated vasoconstriction with diametrically opposing effects; while CXCR4 activation enhanced, ACKR3 activation attenuated α_1_-AR-induced vasoconstriction [7]. Subsequently, we provided evidence that α_1A_-AR preferentially forms heteromeric complexes with CXCR4 and ACKR3 protomers/homodimers, whereas α_1B/D_-AR hetero-oligomerizes with the CXCR4 : ACKR3 heteromer, which appears to be essential for α_1B/D_-AR signalling and function in human vascular smooth muscle cells (hVSMCs) [19–21]. Furthermore, we have shown that simultaneous blockade of CXCR4 and activation of ACKR3 with the synthetic ligand TC14012 result in vasodilatory shock and cardiovascular collapse in normal animals [7]. It appears unlikely, however, that these effects can be attributed exclusively to ACKR3-mediated inhibition of α_1_-AR in vascular smooth muscle, thus suggesting additional interactions between ACKR3 and the vasoactive neurohormonal system. Because interactions between CXCL12 and arginine vasopressin (aVP) have previously been observed in the central nervous system [22,23], we tested whether aVP receptors (AVPRs) cross-talk with ACKR3 and/or CXCR4 in the regulation of intrinsic vascular smooth muscle function. Here, we provide evidence that ACKR3 also regulates AVPR1A signalling and function in VSMC via formation of heteromeric receptor complexes. We show that heteromerization between AVPR1A and ACKR3 facilitates AVPR1A-mediated Gα_q_ signalling and limits aVP-induced β-arrestin 2 recruitment to AVPR1A, and that activation of ACKR3 inhibits aVP-mediated signalling and vasoconstriction. These findings suggest that ACKR3 is a regulator of VSMC function, which controls endogenous and clinically important vasopressor actions.

## 2. Results and discussion

### 2.1. Activation of ACKR3 antagonizes aVP-mediated Gα_q_ signalling and function in vascular smooth muscle

**Figure 1.**
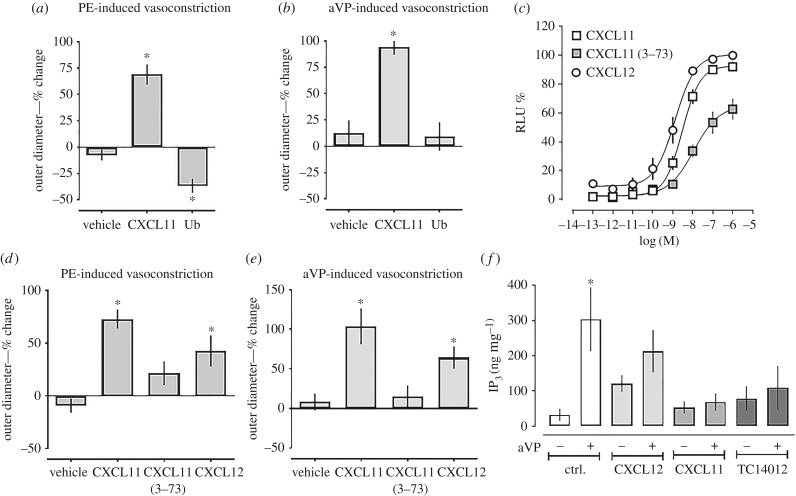
ACKR3 agonists antagonize aVP-mediated Gα_q_ signalling and function in vascular smooth muscle. (*a*,*b*) Pressure myography with rat mesenteric arteries. Arteries were pressurized to 80 mmHg, preconstricted with 2 µM PE (*a*) or 0.5 nM aVP (*b*), followed by the addition of vehicle (*n* = 4) or 10 µM of CXCL11 (*n* = 6) or ubiquitin (*n* = 7). Outer diameter % change: % change in outer diameter after the addition of the CXCR4/ACKR3 ligands. *p < 0.05 versus vehicle. (*c*) β-arrestin 2 recruitment assay (PRESTO-Tango) for ACKR3. HTLA cells were treated with CXCL12, CXCL11 or CXCL11 (3–73). RLU (%): relative luminescence units in % RLU after treatment with 1 µM CXCL12 (= 100%). *n* = 3 independent experiments. (*d*) Pressure myography experiments as in (*a*); PE-induced vasoconstriction. All ACKR3 ligands were tested at a concentration of 10 µM. Vehicle (*n* = 5), CXCL11 (*n* = 7), CXCL11 (3–73) (*n* = 9) and CXCL12 (*n* = 14). *p < 0.05 versus vehicle. (*e*) Pressure myography experiments as in (*b*); aVP-induced vasoconstriction. All ACKR3 ligands were tested at a concentration of 10 µM. Vehicle (*n* = 4), CXCL11 (*n* = 3), CXCL11 (3–73) (*n* = 3) and CXCL12 (*n* = 3). *p < 0.05 versus vehicle. (*f*) hVSMCs were pretreated with either vehicle (ctrl.) or ACKR3 ligands (1 µM, 15 min) and then stimulated with 1 µM aVP for 5 min. IP_3_ production was measured by ELISA. *n* = 4. *p < 0.05 versus vehicle-treated cells.

We used pressure myography to assess whether CXCR4 and/or ACKR3 activation influence aVP-induced constriction of isolated mesenteric resistance arteries. α_1_-AR-induced vasoconstriction upon phenylephrine (PE) stimulation was used as a positive control. In agreement with our previous observations [7], the ACKR3 and CXCR3 agonist CXCL11 antagonized PE-induced vasoconstriction, whereas the CXCR4 agonist ubiquitin, which does not bind to ACKR3 [24], enhanced PE-induced vasoconstriction (figure 1*a*). While ubiquitin did not affect aVP-induced vasoconstriction, CXCL11 also antagonized vasoconstriction upon aVP stimulation (figure 1*b*). To confirm these effects of ACKR3 activation on aVP-induced vasoconstriction, we tested the effects of CXCL12, a CXCR4 and ACKR3 agonist, and of CXCL11 (3–73), an N-terminal truncated form of CXCL11 lacking amino acids 1 and 2, which has been described to show significantly reduced biological activity, when compared with wild-type CXCL11 [25,26]. To compare ACKR3 activity of these ligands, we first measured β-arrestin 2 recruitment to ACKR3 upon ligand stimulation using the PRESTO-Tango cell system [27,28] (figure 1*c*). While the potency of CXCL12 to recruit β-arrestin 2 to ACKR3 was 2.2-fold higher (EC_50_ (95% confidence interval (CI) 1.3 (0.8–2.1) nM) than that of CXCL11 (EC_50_ (95% CI) 2.9 (2.1–4.0) nM, *p* < 0.01), both agonists showed comparable efficacy for β-arrestin 2 recruitment to ACKR3. When compared with CXCL11 and CXCL12, potency and efficacy of CXCL11 (3–73) for β-arrestin 2 recruitment to ACKR3 were significantly reduced (EC_50_ (95% CI) 11 (4–240) nM, top plateau: 65 ± 7% relative luminescence (RLU), *p* < 0.01 for both versus CXCL11 and CXCL12). When tested in pressure myography experiments, CXCL11 and CXCL12 attenuated PE- and aVP-induced vasoconstriction to a similar degree (*p* < 0.05 for vehicle versus CXCL11 and CXCL12; *p* > 0.05 for CXCL11 versus CXCL12), whereas CXCL11 (3–73) did not (figure 1*d*,*e*). The previous findings that CXCL12 binds to ACKR3 with sevenfold to 20-fold higher affinity than CXCL11 are not contradictive to our observations, because maximal biological responses of other GPCRs have been observed at ligand occupancies of only a small fraction of receptors [29–32]. In addition, our findings are consistent with the recent observation that the potency of CXCL12 to induce β-arrestin recruitment to ACKR3 when measured in a bioluminescence resonance energy transfer (BRET)-based assay was twofold higher than that of CXCL11 [30]. The significantly reduced potency and efficacy of truncated CXCL11 (3–73) to activate ACKR3 signalling suggest that its weak agonist activity was insufficient to evoke the functional biological response in intact arteries that we observed with the native ACKR3 agonists.

To test whether the antagonizing effects of ACKR3 are accompanied by corresponding effects on AVPR-mediated Gα_q_ signalling, we measured inositol trisphosphate (IP_3_) production in hVSMC upon aVP stimulation. As shown in figure 1*f*, CXCL11, CXCL12 and TC14012, a synthetic ACKR3 agonist and CXCR4 antagonist [33], inhibited aVP-induced IP_3_ production. These data suggest that ACKR3 activation inhibits AVPR signalling and function in hVSMC.

### 2.2. ACKR3 forms heteromeric complexes with AVPR1A

**Figure 2.**
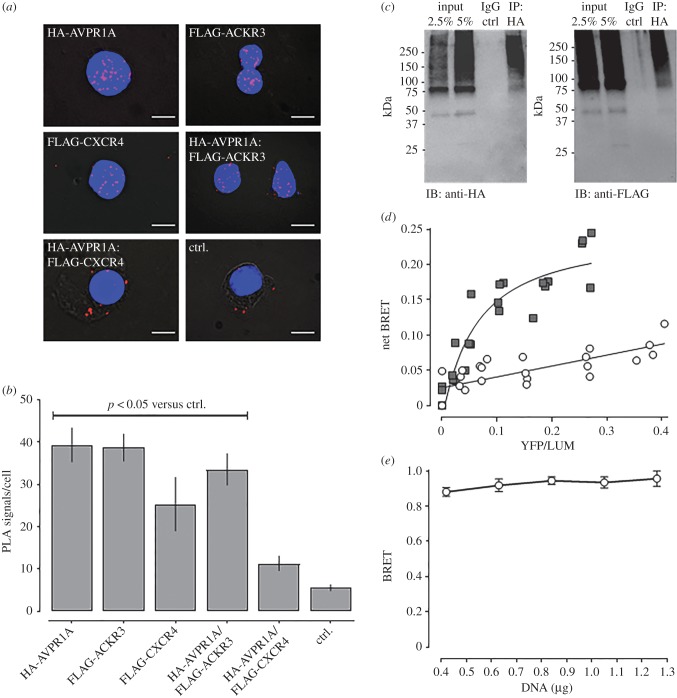
Recombinant ACKR3 and AVPR1A form heteromeric complexes. (*a*) Typical PLA images for the detection of individual receptors and receptor–receptor interactions in HEK293T cells transfected with DNA encoding HA- or FLAG-tagged receptors. Ctrl: cells transfected with pcDNA. Images show merged PLA/DAPI signals acquired from *z*-stack images (*n* = 10; thickness 1 µm, bottom to top). Scale bars, 10 µm. (*b*) Quantification of PLA signals per cell as in (*a*). *n* = 3 independent experiments with *n* = 10 images per condition and experiment. (*c*) HEK293T cells expressing HA-AVPR1A and FLAG-ACKR3 were lysed (input), the lysate was immunoprecipitated (IP) with anti-HA, followed by immunoblotting (IB) to detect HA-AVPR1A (left) and FLAG-ACKR3 (right) in the IP samples. IP control: precipitate after incubation of cell lysates with IgG-coupled resin. (*d*,*e*) Intermolecular BRET assays. Cells were co-transfected with AVPR1A-hRluc plus EYFP (open circles) or ACKR3-EYFP (grey squares) at various acceptor : donor ratios (*d*) and with increasing amounts of AVPR1A-hRluc and ACKR3-EYFP at a constant ratio of 1 : 10 (*e*).

We have shown previously that α_1_-ARs form hetero-oligomeric complexes with ACKR3 and CXCR4 in hVSMC, through which α_1B/D_-AR signalling and function are regulated [19–21]. Thus, we tested whether CXCR4 and ACKR3 may also form hetero-oligomeric complexes with AVPR1A, the receptor subtype which is responsible for the vasopressor action of aVP in VSMC [34]. We first co-expressed recombinant FLAG-tagged CXCR4 (FLAG-CXCR4) or FLAG-ACKR3 with human influenza haemagglutinin (HA)-tagged AVPR1A (HA-AVPR1A) in HEK293T cells and performed proximity ligation assays (PLAs) with anti-HA and anti-FLAG to visualize and quantify individual receptors and receptor–receptor interactions at single-molecule resolution [35]. Figure 2*a* shows representative PLA images for the detection of HA- and FLAG-tagged receptors and receptor–receptor interactions, and figure 2*b* shows the quantification of the corresponding PLA signals from three independent experiments. We observed positive signals corresponding to HA-AVPR1A : FLAG-ACKR3 interactions. By contrast, the number of PLA signals for HA-AVPR1A : FLAG-CXCR4 interactions was not significantly different from that of PLA signals in negative control experiments. To confirm the observation that HA-AVPR1A interacts with FLAG-ACKR3 in PLA experiments, we immunoprecipitated HA-AVPR1A with an anti-HA antibody and then performed western blot experiments with anti-HA and anti-FLAG to detect HA-AVPR1A and FLAG-ACKR3, respectively. As shown in figure 2*c* (left), when the cell lysate (input) was probed with anti-HA, we observed a band below 50 kDa and numerous bands in the high-molecular-mass range, which probably corresponds to the HA-AVPR1A monomer with the majority of receptors migrating as aggregates. The latter were also detectable in the HA-immunoprecipitate, but not in the IgG-immunoprecipitate. When probed with anti-FLAG (figure 2*c*, right), a similar pattern was detectable in the cell lysate, and both a faint band below 50 kDa and high-molecular aggregates were detectable in the HA-immunoprecipitate but not in the IgG-immunoprecipitate, indicating that FLAG-immunoreactivty could be precipitated with anti-HA.

Next, we used plasmids encoding AVPR1A-hRluc (*Renilla reniformis*), enhanced yellow fluorescent protein (EYFP) and ACKR3-EYFP for intermolecular BRET experiments (figure 2*d*,*e*). In cells expressing EYFP and AVPR1A-hRluc, the BRET signal was low and increased linearly with increasing acceptor : donor ratios, which is consistent with a non-specific bystander BRET signal (figure 2*d*). The BRET signals in cells expressing ACKR3-EYFP and AVPR1A-hRluc showed a hyperbolic progression with increasing acceptor : donor ratios (figure 2*d*), and the BRET signal was independent of the concentrations of BRET partners when tested at a fixed acceptor : donor ratio (figure 2*e*), indicating constitutive heteromerization [36].

**Figure 3.**
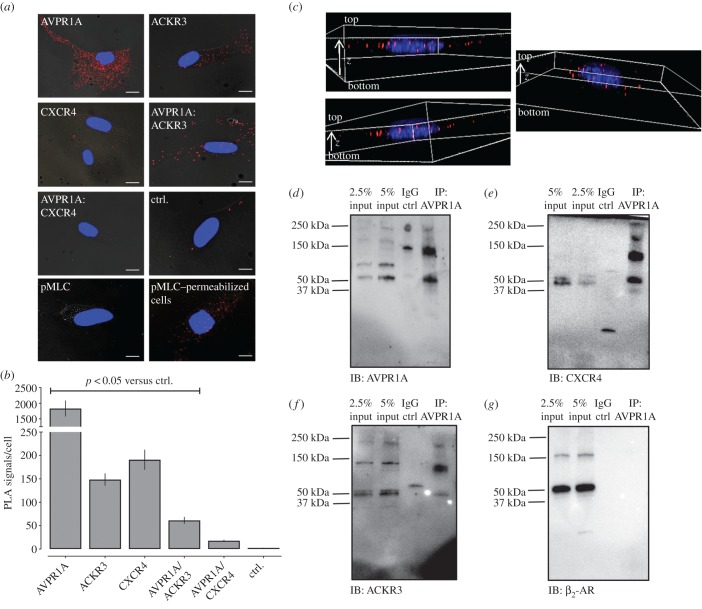
ACKR3 and AVPR1A form heteromeric complexes in hVSMCs. (*a*) Representative PLA images for the detection of individual receptors, receptor–receptor complexes and pMLC2 in hVSMC. PLA for pMLC2 was performed in non-permeabilized cells (pMLC2, bottom left) and permeabilized cells (pMLC2, permeabilized cells, bottom right). All other PLAs were performed in non-permeabilized cells. Ctrl: omission of one primary antibody. Images show merged PLA/DAPI signals acquired from *z*-stack images (*n* = 10; thickness 1 µm, bottom to top). Scale bars, 10 µm. (*b*) Quantification of PLA signals per cell as in (*a*). *n* = 4 independent experiments with *n* = 10 images per condition and experiment. (*c*) Three-dimensional representations of ACKR3 : AVPR1A interactions in hVSMC. Deconvolved images were generated from *z*-stack images (*n* = 20; thickness: 0.5 µm, bottom to top). Images show merged PLA/DAPI signals. (*d–g*) hVSMCs were lysed (=input) and AVPR1A was immunoprecipitated (IP) followed by immunoblotting (IB) to detect AVPR1A (*d*), CXCR4 (*e*), ACKR3 (*f*) and *β*_2_-AR (*g*) in the IP samples. IP control: precipitate after incubation of cell lysates with IgG-coupled resin. Images are representative of *n* = 4 independent experiments.

As PLA, co-immunoprecipitation and BRET assays collectively suggested that recombinant AVPR1A heteromerizes with ACKR3 when co-expressed, we tested whether such interactions are also detectable for endogenously expressed receptors using PLA to detect receptor–receptor interactions in hVSMC. The anti-GPCR antibodies that were used in PLA are directed against extracellular domains of the receptors and have been validated for sufficient selectivity for their GPCR targets previously [19,21,37]. Figure 3*a* shows representative PLA images and figure 3*b* shows the quantification of PLA signals for individual receptors and receptor interactions from four independent experiments. In line with our findings on recombinant receptors, we observed positive PLA signals for endogenous ACKR3 : AVPR1A interactions, whereas signals for CXCR4 : AVPR1A interactions were indistinguishable from negative control experiments. Furthermore, we observed that PLA signals for phosphorylated (Ser-19) myosin light chain (pMLC) 2 (figure 3*a*, bottom left) were indistinguishable from negative controls in cells that were not permeabilized, whereas positive PLA signals were detectable in cells after permeabilization (figure 3*a*, bottom right). In line with our previous findings [19], this observation is consistent with the intracellular localization of pMLC2 and demonstrates that antibodies do not reach intracellular compartments when PLA is performed in non-permeabilized cells. This indicates that the PLA signals for individual receptors and receptor interactions that were obtained with anti-GPCR antibodies directed against extracellular receptor domains in non-permeabilized hVSMCs are localized on the cell surface under our experimental conditions. This assumption is supported by three-dimensional reconstruction of the PLA signals from deconvolved *z*-stack images, which showed that most PLA signals for ACKR3 : AVPR1A interactions are localized in a single plane (figure 3*c*).

To confirm these observations, we performed immunoprecipitation experiments with hVSMC. AVPR1A could be precipitated with anti-AVPR1A (figure 3*d*). ACKR3 and CXCR4 were detectable in the AVPR1A immunoprecipitate (figure 3*e*,*f*). β_2_-AR, which was used as a negative control, could not be detected (figure 3*g*). The combination of the findings that positive PLA, co-immunoprecipitation and BRET signals were detectable for recombinant ACKR3 : AVPR1A interactions, that positive PLA signals for endogenous ACKR3 : AVPR1A interactions were detectable and that endogenous ACKR3 could be co-immunoprecipitated with AVPR1A indicates that both receptors form heteromeric complexes in hVSMC. As CXCR4 is known to form heteromeric complexes with ACKR3 [10,11,19], the findings that CXCR4 was detectable in AVPR1A immunoprecipitates, despite the lack of significant PLA signals for CXCR4 : AVPR1A interactions, could suggest that CXCR4 exists within the same plasma membrane microdomains as ACKR3 and AVPR1A, but is not in sufficient proximity to AVPR1A to permit direct interactions. Alternatively, it appears possible that the abundance of CXCR4 : AVPR1A interactions is too low to be discriminated with confidence by PLA under our experimental conditions.

### 2.3. Depletion of ACKR3 : AVPR1A heteromers by ACKR3 knockdown increases AVPR1A : CXCR4 heteromerization and inhibits aVP-induced Gα_q_ signalling

**Figure 4.**
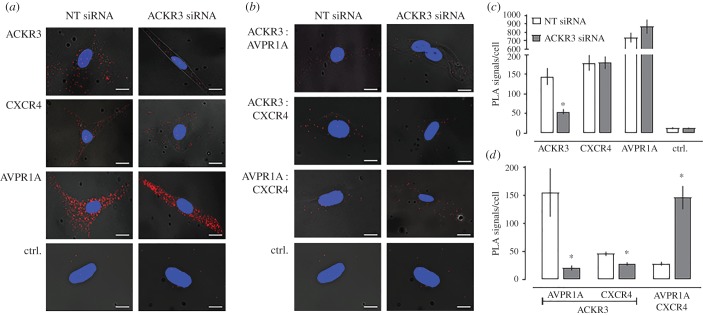
ACKR3 gene silencing reduces ACKR3 : AVPR1A and ACKR3 : CXCR4 heteromers and increases AVPR1A : CXCR4 interactions in hVSMCs. (*a*,*b*) Representative PLA images for the detection of individual receptors (*a*) and receptor–receptor interactions (*b*) in hVSMC after incubation with NT or ACKR3 siRNA. Ctrl: omission of one primary antibody. Images show merged PLA/4′,6-diamidino-2-phenylindole dihydrochloride (DAPI) signals acquired from *z*-stack images (*n* = 10; thickness 1 µm, bottom to top). (*c*,*d*) Quantification of PLA signals per cell for the detection of individual receptors (*c*) and receptor–receptor interactions (*d*) as in (*a*,*b*). *n* = 4 independent experiments with *n* = 10 images per condition and experiment. *p < 0.05 versus cells incubated with NT-siRNA.

**Figure 5.**
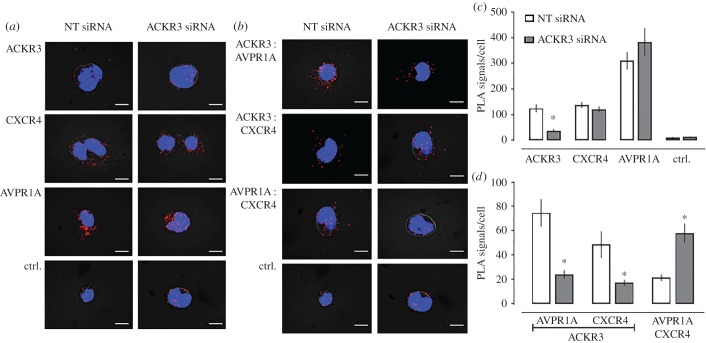
ACKR3 gene silencing reduces ACKR3 : AVPR1A and ACKR3 : CXCR4 heteromers and increases AVPR1A : CXCR4 interactions in A7r5 cells. (*a*,*b*). Representative PLA images for the detection of individual receptors (*a*) and receptor–receptor interactions (*b*) in A7r5 cells after incubation with NT or ACKR3 siRNA. Ctrl: omission of one primary antibody. Images show merged PLA/4′,6-diamidino-2-phenylindole dihydrochloride (DAPI) signals acquired from *z*-stack images (*n* = 10; thickness 1 µm, bottom to top). (*c*,*d*) Quantification of PLA signals per cell for the detection of individual receptors (*c*) and receptor–receptor interactions (*d*) as in (*a*,*b*). *n* = 6 independent experiments with *n* = 10 images per condition and experiment. *p < 0.05 versus cells incubated with NT-siRNA.

To evaluate functional consequences of ACKR3 : AVPR1A heteromerization on AVPR1A signalling in hVSMC, we depleted ACKR3 : AVPR1A heteromers by ACKR3 knockdown with siRNA. Figure 4 shows typical PLA images for the detection of individual receptors (figure 4*a*), receptor–receptor interactions (figure 4*b*) and the quantification of the number of corresponding PLA signals from four independent experiments (figure 4*c*,*d*). When compared with hVSMC after incubation with non-targeting (NT) siRNA, PLA signals for ACKR3 were reduced by more than 60% after incubation with ACKR3 siRNA (figure 4*c*). PLA signals for CXCR4 and AVPR1A were indistinguishable in hVSMC incubated with NT and ACKR3 siRNA (figure 4*c*). When PLA was performed to detect receptor–receptor interactions (figure 4*d*), we observed that signals corresponding to ACKR3 : AVPR1A and ACKR3 : CXCR4 heteromers in hVSMC incubated with ACKR3 siRNA were reduced by 80% and 50%, respectively, when compared with hVSMC incubated with NT-siRNA. Surprisingly, in hVSMC incubated with ACKR3 siRNA, PLA signals corresponding to CXCR4 : AVPR1A interactions increased to 510% of PLA signals in hVSMC incubated with NT-siRNA. To confirm these observations, we repeated the siRNA knockdown experiments in the rat vascular smooth muscle cell line A7r5. Figure 5 shows typical PLA images for the detection of individual receptors (figure 5*a*), receptor–receptor interactions (figure 5*b*) and the quantification of the number of corresponding PLA signals from six independent experiments (figure 5*c*,*d*). As in hVSMC, we also observed positive PLA signals for ACKR3 : AVPR1A heteromers in A7r5 cells. While rat and human ACKR3 show 93% sequence identity, rat and human AVPR1A share only 79% sequence identity [38]. The positive PLA signals for ACKR3 : AVPR1A heteromers in A7r5 cells indicate that these differences between the rat and human AVPR1A sequences do not affect interactions between the receptor partners. Similar to hVSMC, we observed that ACKR3 depletion in A7r5 cells by siRNA knockdown reduced PLA signals for ACKR3 : AVPR1A and ACKR3 : CXCR4 interactions proportional to the degree of ACKR3 knockdown, but increased PLA signals for CXCR4 : AVPR1A interactions to 276% of PLA signals in A7r5 cells incubated with NT-siRNA. These findings suggest that ACKR3 hinders CXCR4 : AVPR1A interactions, which occur after depletion of ACKR3 from the cell surface. Such a behaviour would be in agreement with previous observations, indicating that hetero-oligomeric receptor complexes within the plasma membrane exist in a dynamic equilibrium, in which interference with heteromerization between two receptors shifts the patterns of receptor hetero-oligomerization within the entire receptor network towards a new equilibrium, leading to newly formed heteromeric receptor complexes [21,39–41]. Furthermore, these observations imply that the insignificant number of PLA signals for CXCR4 : AVPR1A interactions that were detectable in hVSMC probably corresponds to a very low abundance of CXCR4 : AVPR1A heteromers.

**Figure 6.**
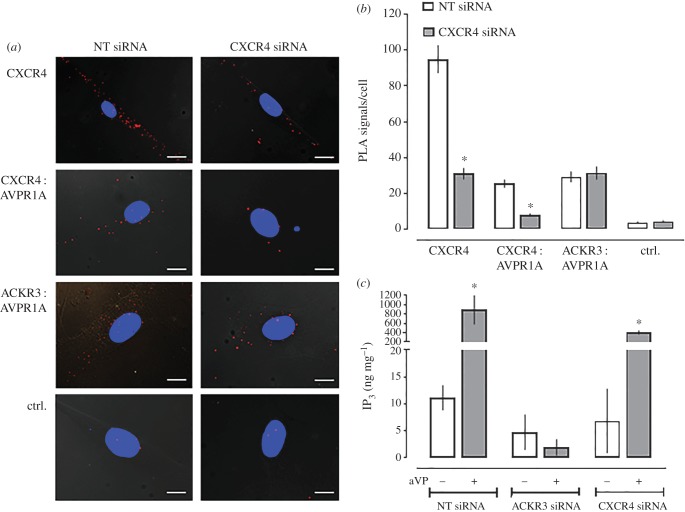
ACKR3 gene silencing inhibits aVP-mediated Gα_q_ signalling, whereas CXCR4 gene silencing does not affect ACKR3 : AVPR1A heteromerization and aVP-mediated Gα_q_ signalling. (*a*) Representative PLA images for the detection of CXCR4 and receptor–receptor interactions in hVSMC after incubation with NT or CXCR4 siRNA. Ctrl: omission of one primary antibody. Images show merged PLA/4′,6-diamidino-2-phenylindole dihydrochloride (DAPI) signals acquired from *z*-stack images (*n* = 10; thickness 1 µm, bottom to top). Scale bars, 10 µm. (*b*) Quantification of PLA signals per cell as in (*a*). *n* = 4 independent experiments with *n* = 10 images per condition and experiment. *p < 0.05 versus cells incubated with NT-siRNA. (*c*) IP_3_ production of hVSMC incubated with NT, ACKR3 or CXCR4 siRNA upon stimulation with vehicle (−) or 1 µm aVP (+) for 5 min. *n* = 4 independent experiments. *p < 0.05 versus vehicle.

To assess the effect of CXCR4 knockdown on the formation of heteromeric complexes between AVPR1A and ACKR3 or CXCR4 in hVSMC, we then silenced CXCR4 with siRNA. Figure 6*a* shows representative PLA images for the detection of CXCR4 and of CXCR4 : AVPR1A and ACKR3 : AVPR1A heteromers in hVSMC incubated with NT or CXCR4 siRNA, and figure 6*b* shows the quantification of corresponding PLA signals from four independent experiments. When compared with hVSMC incubated with NT-siRNA, PLA signals for CXCR4 were reduced by 70% after incubation with CXCR4 siRNA. While PLA signals for CXCR4 : AVPR1A interactions decreased to the same degree, CXCR4 silencing did not affect the number of PLA signals for ACKR3 : AVPR1A interactions.

These findings suggest that AVPR1A preferentially forms heteromeric complexes with ACKR3 and CXCR4 protomers or homodimers, but not with CXCR4 : ACKR3 heteromers. This interaction pattern is similar to the interaction pattern that we previously observed for α_1A_-AR : CXCR4 and α_1A_-AR : ACKR3 heteromers, and distinct from the interaction pattern of α_1B/D_-AR, which preferentially form hetero-oligomeric complexes with the CXCR4 : ACKR3 heteromer [21].

Measurements of aVP-induced IP_3_ production in hVSMC after ACKR3 and CXCR4 silencing with siRNA are shown in figure 6*c*. While there were no significant differences in aVP-induced IP_3_ production in hVSMC incubated with NT and CXCR4 siRNA, aVP-induced IP_3_ production in hVSMC was abolished after incubation with ACKR3 siRNA. These findings indicate that heteromerization between AVPR1A and ACKR3 is required for AVPR1A signalling in hVSMC, which is similar to the requirement of hetero-oligomerization between α_1B/D_-AR and the CXCR4 : ACKR3 heteromeric complex for α_1B/D_-AR signalling in hVSMC that we described previously [21]. While ACKR3 within the ACKR3 : AVPR1A complex may serve to stabilize AVPR1A in a configuration capable of coupling to Gα_q_, it appears also possible that the formation of AVPR1A : CXCR4 heteromers after ACKR3 silencing could be responsible for the loss of aVP-induced IP_3_ production. The latter would imply that CXCR4 within the heteromeric AVPR1A : CXCR4 complex hinders AVPR1A signalling upon agonist stimulation.

### 2.4. Transmembrane domain-derived peptide analogues of ACKR3 interfere with AVPR1A : ACKR3 heteromerization and aVP-induced Gα_q_ signalling

**Figure 7.**
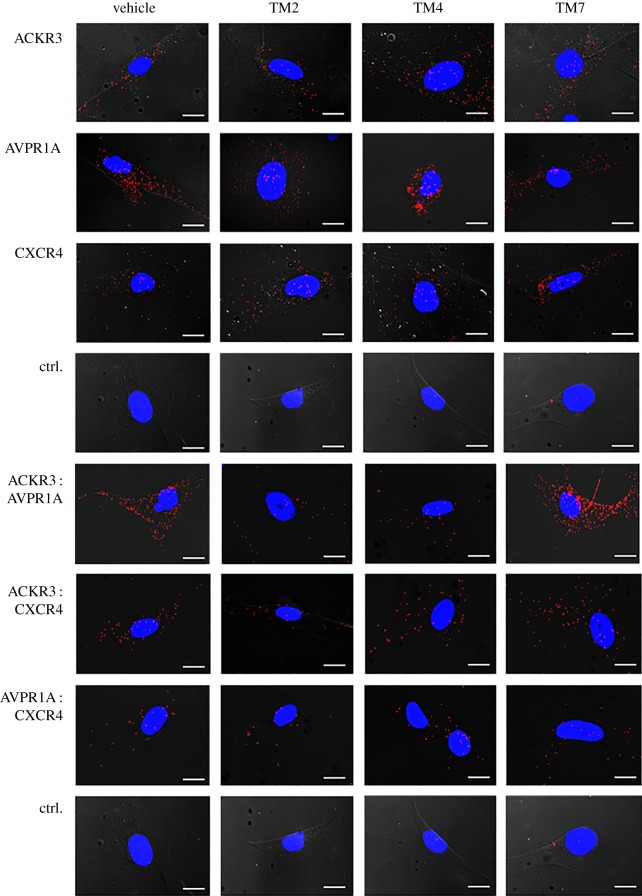
ACKR3-derived TM peptide analogues disrupt ACKR3 : AVPR1A and ACKR3 : CXCR4 heteromeric complexes. hVSMCs were incubated with vehicle, TM2, TM4 or TM7 (10 µM, 30 min at 37°C), washed and fixed for PLA. Typical PLA images for the detection of individual receptors or receptor–receptor complexes are shown. Ctrl: omission of one primary antibody. Images show merged PLA/4′,6-diamidino-2-phenylindole dihydrochloride (DAPI) signals acquired from *z*-stack images (*n* = 10; thickness 1 µm, bottom to top). Scale bars, 10 µm.

**Figure 8.**
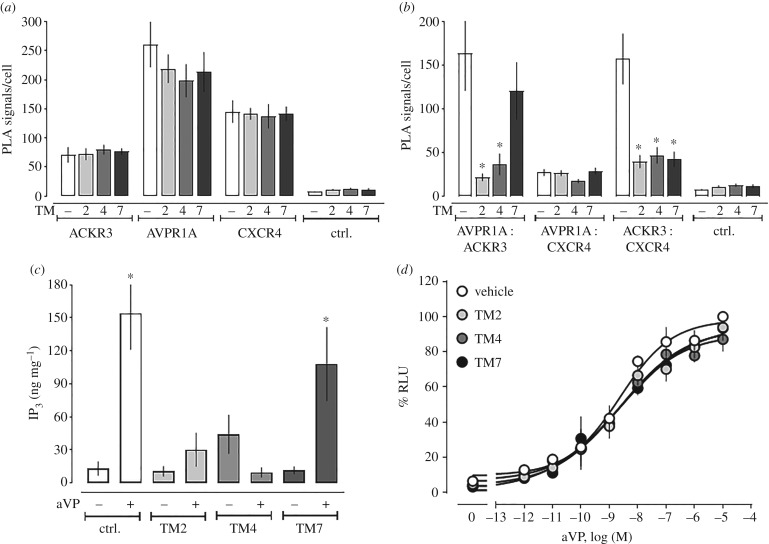
ACKR3-derived TM peptide analogues interfere with ACKR3 : AVPR1A and ACKR3 : CXCR4 heteromerization and inhibit aVP-induced Gα_q_ signalling in hVSMC. (*a*,*b*) Quantification of PLA signals per cell as in figure 7 for individual receptors (*a*) and receptor–receptor interactions (*b*). *n* = 4 independent experiments with *n* = 10 images per condition and experiment. *p < 0.05 versus cells incubated with vehicle. (*c*) IP_3_ production was measured in hVSMC pretreated with vehicle or TM2/4/7 peptide analogues (10 µM, 30 min at 37°C) and then stimulated with either vehicle (−) or aVP (1 µM, 5 min) (+). *n* = 4 independent experiments. *p < 0.05 versus vehicle-treated cells. (*d*) β-arrestin 2 recruitment assay (PRESTO-Tango) for AVPR1A. HTLA cells expressing FLAG-AVPR1A-Tango were incubated with either vehicle or ACKR3 TM2/4/7 (10 µM, 30 min at 37°C) and then stimulated with aVP overnight. RLU (%): relative luminescence units in % RLU after treatment with 10 µM aVP (=100%). *n* = 3 independent experiments.

TM-derived peptide analogues of GPCRs have previously been used to disrupt heteromerization and alter receptor function [19–21,42–44]. Thus, we tested whether TM2, TM4 and TM7 peptide analogues of ACKR3 interfere with AVPR1A : ACKR3 heteromerization. Representative images from PLA for the detection of individual receptors and receptor–receptor interactions in hVSMC incubated with vehicle or TM peptides are shown in figure 7, and the quantifications of corresponding PLA signals from three independent experiments are shown in figure 8*a*,*b*. The TM peptides did not affect PLA signals for AVPR1A, ACKR3 or CXCR4 (figure 8*a*). Furthermore, none of the TM peptides affected PLA signals for AVPR1A : CXCR4 interactions (figure 8*b*). While the TM2 and TM4 peptides reduced PLA signals for AVPR1A : ACKR3 interactions, all TM peptide analogues reduced PLA signals for CXCR4 : ACKR3 interactions. These findings confirm our previous observations on the effects of the TM peptides on CXCR4 : ACKR3 heteromerization and mimic their effects on the formation of heteromeric complexes between α_1B_-AR and ACKR3 [21]. The latter suggests that α_1B_-AR and AVPR1A may form heteromeric complexes via similar interaction sites on ACKR3.

The effects of the TM peptide analogues on aVP-induced IP_3_ production in hVSMC are shown in figure 8*c*. Consistent with their effects on ACKR3 : AVPR1A heteromerization, we observed that the TM2 and TM4 peptide analogues inhibited aVP-induced IP_3_ production in hVSMC, whereas the TM7 peptide analogue was ineffective. To address the possibility that the TM peptides directly affect AVPR1A function, we performed β-arrestin 2 recruitment assays for AVPR1A. As shown in figure 8*d*, none of the peptides affected β-arrestin 2 recruitment to AVPR1A upon aVP stimulation. These findings are in agreement with the loss of aVP-responsiveness of hVSMC after ACKR3 silencing, and further suggest that heteromerization between AVPR1A and ACKR3 is required for AVPR1A signalling in hVSMC. Because the TM2 and TM4 peptide analogues did not increase AVPR1A : CXCR4 interactions, formation of such interactions cannot account for the loss of aVP-responsiveness. This supports the concept that ACKR3 within the ACKR3 : AVPR1A complex serves to stabilize AVPR1A in a functional configuration.

### 2.5. The AVPR1A : ACKR3 heteromeric complex modulates β-arrestin recruitment to each receptor partner and shows asymmetrical β-arrestin cross-recruitment upon agonist stimulation

**Figure 9.**
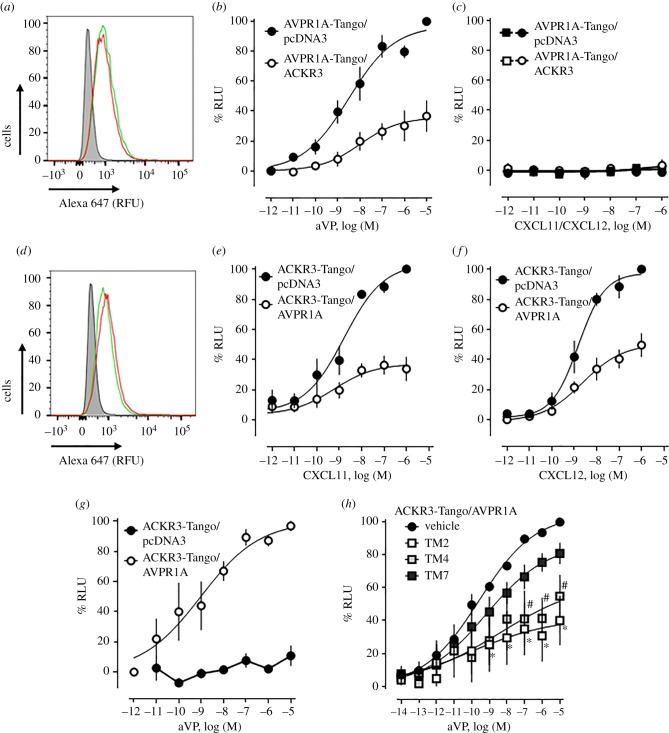
β-arrestin 2 recruitment at the ACKR3 : AVPR1A heteromeric complex. (*a–c*) HTLA cells were co-transfected with 0.75 µg of DNA for FLAG-AVPR1A-TANGO plus 0.75 µg of pcDNA3 or HA-ACKR3. (*a*) Measurement of FLAG-AVPR1A-Tango expression by flow cytometry. Cells were labelled with Alexa Fluor 647 anti-FLAG. Grey area: unstained cells. Red line: cells transfected with FLAG-AVPR1A-TANGO plus pcDNA. Green line: cells transfected with FLAG-AVPR1A-TANGO/HA-ACKR3. RFU: relative fluorescence units. Data are representative of *n* = 3 independent experiments. (*b*,*c*) β-arrestin 2 recruitment assay (PRESTO-Tango) for AVPR1A. Cells were stimulated with aVP (*b*), CXCL11 (*c*) and CXCL12 (*c*). Black symbols: cells transfected with FLAG-AVPR1A-TANGO/pcDNA3; white symbols: cells transfected with FLAG-AVPR1A-TANGO/HA-ACKR3. *n* = 3 independent experiments. (*d–g*) HTLA cells were co-transfected with 0.75 µg of DNA for FLAG-ACKR3-TANGO plus 0.75 µg of pcDNA3 or HA-AVPR1A. (*d*) Measurement of FLAG-ACKR3-Tango expression by flow cytometry. Cells were labelled with Alexa Fluor 647 anti-FLAG. Grey area: unstained cells. Red line: cells transfected with FLAG-ACKR3-TANGO plus pcDNA3. Green line: cells transfected with FLAG-ACKR3-TANGO/HA-AVPR1A. RFU: relative fluorescence units. Data are representative of *n* = 3 experiments. (*e–g*) β-arrestin 2 recruitment assay (PRESTO-Tango) for ACKR3. Cells were stimulated with CXCL11 (*e*), CXCL12 (*f*) and aVP (*g*). Black circles: cells transfected with FLAG-ACKR3-TANGO/pcDNA3; white circles: cells transfected with FLAG-ACKR3-TANGO/HA-AVPR1A. *n* = 3 independent experiments. (*h*) β-arrestin 2 recruitment assay (PRESTO-Tango) with HTLA cells co-expressing FLAG-AVPR1A-Tango/HA-ACKR3. Cells were treated with vehicle or TM2/4/7 peptides (10 µM, 30 min at 37°C) and then stimulated with aVP. *n* = 3 independent experiments. Black circle: vehicle. Open squares: TM2. Light grey squares: TM4. Dark grey squares: TM7. *n* = 4 independent experiments.

We used the PRESTO-Tango cell system to evaluate whether heteromerization between AVPR1A and ACKR3 also modulates β-arrestin 2 recruitment upon agonist stimulation. We first co-expressed FLAG-AVPR1A-Tango with pcDNA3 or HA-ACKR3, confirmed comparable FLAG-AVPR1A-Tango expression by flow cytometry (figure 9*a*) and determined the dose–response for β-arrestin 2 recruitment upon agonist stimulation (figure 9*b*). In cells co-expressing AVPR1A-Tango/pcDNA3, aVP induced β-arrestin 2 recruitment with an EC_50_ (95% CI) of 2.5 (0.8–8.8) nM and top plateau was 97 ± 7 RLU%. While the potency of aVP to recruit β-arrestin 2 in cells co-expressing AVPR1A-Tango/ACKR3 was similar to cells co-expressing AVPR1A-Tango/pcDNA3 (EC_50_ (95% CI): 6.1 (0.7–58) nM, *p* > 0.05 versus AVPR1A-Tango/pcDNA3), the efficacy of aVP was significantly reduced (top plateau: 32 ± 4 RFU%, *p* < 0.001 versus AVPR1A-Tango/pcDNA3). CXCL11 and CXCL12 did not induce β-arrestin 2 recruitment to AVPR1A-Tango in the presence or absence of ACKR3 (figure 9*c*). When cells co-expressing similar levels of ACKR3-Tango plus pcDNA3 or AVPR1A (figure 9*d*) were tested, we observed that the presence of AVPR1A significantly reduced the efficacy of CXCL11 (figure 9*e*) and CXCL12 (figure 9*f*) to recruit β-arrestin 2 to ACKR3-Tango, without affecting the potency of the ACKR3 agonists. While aVP stimulation did not induce β-arrestin 2 recruitment to ACKR3-Tango when co-expressed with pcDNA3, aVP induced β-arrestin 2 recruitment to ACKR3-Tango when co-expressed with AVPR1A (figure 9*g*).

The effects of the TM peptide analogues of ACKR3 on aVP-induced recruitment of β-arrestin 2 to ACKR3-Tango in cells co-expressing ACKR3-Tango and AVPR1A are shown in figure 9*h*. When compared with vehicle-treated cells, the TM2 and TM4 peptide analogues significantly reduced the efficacy of aVP to induce β-arrestin 2 recruitment, whereas the TM7 peptide did not show significant effects. Recently, we reported that the TM2 peptide analogue shows the pharmacological behaviour of a competitive antagonist for β-arrestin 2 recruitment to ACKR3-Tango upon agonist stimulation, whereas the TM7 peptide analogue was inactive; the TM4 peptide exhibited a behaviour similar to the TM2 peptide, but this effect did not reach statistical significance [21]. In the present study, the TM2 and TM4 peptides showed the pharmacological behaviour of non-competitive antagonists, which inhibited aVP-induced β-arrestin 2 recruitment to ACKR3-Tango with comparable efficacy. This behaviour is consistent with the notion that interference of the TM2/4 peptides with ACKR3 : AVPR1A heteromerization is the main mechanism underlying their inhibitory effects on aVP-induced β-arrestin 2 recruitment to ACKR3-Tango in cells co-expressing ACKR3-Tango and AVPR1A.

Our observation that the presence of ACKR3 reduces aVP-induced β-arrestin 2 recruitment to AVPR1A-Tango and that the presence of AVPR1A enables β-arrestin 2 recruitment to ACKR3-Tango upon aVP stimulation indicates that AVPR1A activation within the heteromeric AVPR1A : ACKR3 complex leads to β-arrestin 2 recruitment to both receptor partners. CXCL11 and CXCL12, however, failed to cross-recruit β-arrestin 2 to AVPR1A-Tango in the presence of ACKR3. These findings imply that ACKR3 within the heteromeric receptor complex attenuates β-arrestin 2 recruitment to AVPR1A via allosteric interactions. In combination with the observed effects of AVPR1A : ACKR3 heteromerization on aVP-induced Gα_q_-mediated signalling events, these data suggest that ACKR3 within the AVPR1A : ACKR3 heteromer controls the balance between AVPR1A-mediated Gα_q_ and β-arrestin signalling. Furthermore, our findings demonstrate asymmetrical agonist-induced cross-regulation of ACKR3 by AVPR1A within the heteromeric receptor complex. Such pharmacological behaviour of the AVPR1A : ACKR3 heteromeric complex is similar to the signalling behaviour of other GPCR heteromers, for which ligand-induced symmetrical and asymmetrical cross-activation and cross-inhibition of various signalling read-outs have previously been described [45–48].

### 2.6. AVPR1A and ACKR3 co-internalize upon agonist binding

**Figure 10.**
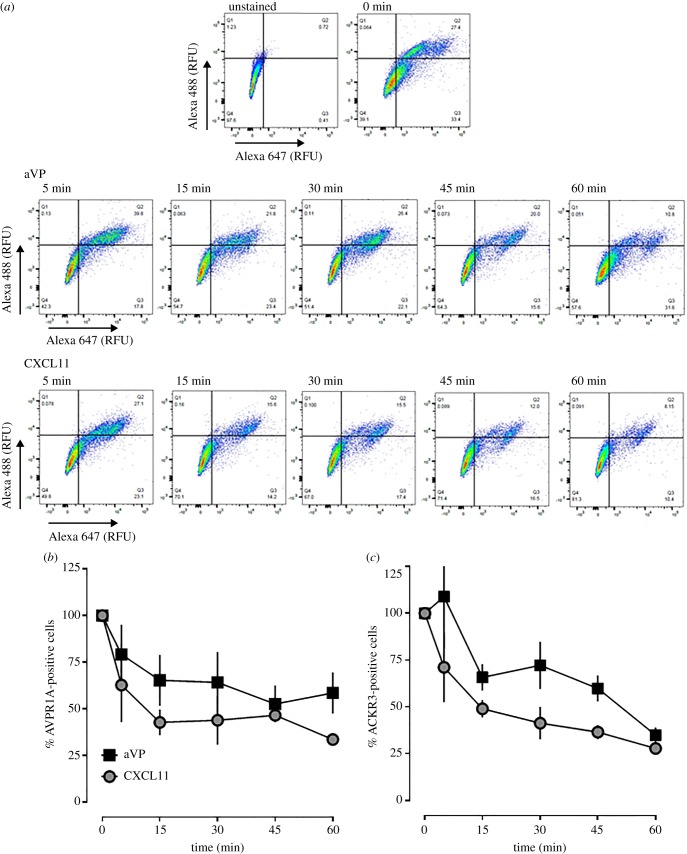
AVPR1A and ACKR3 co-internalize upon agonist stimulation in hVSMC. (*a*) hVSMCs were treated with 1 µM aVP or CXCL11 for up to 60 min, stained at 4°C with rabbit anti-AVPR1A/donkey anti-rabbit Alexa Fluor 647 and mouse anti-ACKR3/goat anti-mouse Alexa Fluor 488 and analysed for receptor expression via flow cytometry. RFU: relative fluorescence units. The horizontal and vertical lines show the gating thresholds for ACKR3 (Alexa 488) and AVPR1A (Alexa 647). (*b*) Quantification of AVPR1A-positive cells after incubation with aVP and CXCL11, as in (*a*). *n* = 3 independent experiments. (*c*) Quantification of ACKR3-positive cells after incubation with aVP and CXCL11, as in (*a*). *n* = 3 independent experiments.

Agonist-induced β-arrestin recruitment to AVPR1A and ACKR3 is known to lead to the reduction of receptor cell surface expression levels, either via induction of receptor internalization or inhibition of receptor recycling [9,49,50]. As activation of recombinant AVPR1A cross-recruited β-arrestin 2 to ACKR3-Tango, we tested whether endogenous AVPR1A and ACKR3 in hVSMC co-internalize upon agonist stimulation. Thus, we stimulated hVSMC with aVP or CXCL11 and quantified AVPR1A and ACKR3 cell surface expression via double-immunofluorescence staining by flow cytometry. Figure 10*a* shows representative two-dimensional scatter plots for the detection of both receptors over a 60-minute time period after stimulation of hVSMC with aVP or CXCL11, and figure 10*b*,*c* shows the quantification of receptor cell surface expression from three independent experiments. aVP and CXCL11 stimulation of hVSMC time-dependently reduced expression levels of both AVPR1A and ACKR3. The time course and the degree of receptor depletion from the cell surface were comparable for both agonists, indicating symmetrical agonist-induced co-internalization of AVPR1A and ACKR3. The finding that AVPR1A and ACKR3 co-internalize upon aVP stimulation matches well with the observed aVP-induced β-arrestin 2 cross-recruitment to ACKR3 within the AVPR1A : ACKR3 complex. CXCL11, however, failed to cross-recruit β-arrestin 2 to recombinant AVPR1A-Tango, yet induced co-internalization of both endogenous receptors. Thus, it appears possible that β-arrestin recruitment to only one of the two receptor partners within the heteromeric complex is sufficient to induce receptor co-internalization.

Taken together, our observations that endogenous AVPR1A and ACKR3 are in close proximity in hVSMC, that the association between endogenous AVPR1A and ACKR3 can be disrupted with selective ACKR3-derived TM peptide analogues without altering expression levels of the individual receptors, and that interference with the association between AVPR1A and ACKR3 in hVSMC alters receptor function fulfil recently proposed criteria for GPCR heteromers in native tissues [51]. While we currently cannot provide direct and unequivocal evidence for the localization of AVPR1A : ACKR3 heteromers on the plasma membrane, our finding that antibodies do not reach intracellular compartments in PLA when cells are not permeabilized, along with the observed changes in AVPR1A function upon exposure of cells to the extracellular agonist after disruption of the AVPR1A : ACKR3 complexes, strongly suggests that these receptor heteromers are located on the plasma membrane. This notion is supported by our findings in expression systems, which provide additional mechanistic insights into molecular events at the AVPR1A : ACKR3 heteromer and demonstrate that heteromerization changes the biochemical fingerprint of each receptor partner [52]. We currently cannot comment on the physiological consequences of AVPR1A : ACKR3 heteromerization on ACKR3-mediated effects on cell function due to the lack of appropriate read-outs that are characteristic for ACKR3 and independent of CXCR4. Similarly, the possible roles of AVPR1A : CXCR4 heteromers, which occur after ACKR3 depletion, remain to be determined. Our findings, however, indicate that AVPR1A : ACKR3 complexes are essential for AVPR1A function in vascular smooth muscle and suggest that ACKR3 activation within the heteromeric complex attenuates aVP-mediated vasoconstriction.

We have shown previously that hetero-oligomerization between α_1B/D_-AR and the ACKR3 : CXCR4 heteromer is essential for α_1B/D_-AR function in vascular smooth muscle and that ACKR3 activation attenuates α_1_-AR-mediated vasoconstriction and α_1B/D_-AR signalling in hVSMC [19–21]. In combination with the findings of the present study, these data support the concept that ACKR3 functions as a critical regulator vascular smooth muscle function: ACKR3 in the agonist-free conformation facilitates Gα_q_ coupling of α_1_-AR and AVPR1A via direct physical interactions within the heteromeric complex. In the agonist-bound conformation, ACKR3 inhibits G protein coupling of the receptor partners and induces internalization of the heteromeric receptor complex.

Our findings provide a molecular mechanism for the previously described effects of synthetic ACKR3 ligands on blood pressure regulation in animals [4,7] and for interactions between the innate immune and vasoactive neurohormonal systems. This function of ACKR3 offers a mechanistic basis for the clinical observation that systemic levels of CXCL12, which preferentially acts as an ACKR3 agonist in VSMC [7], are significantly elevated in patients with sepsis and septic shock; the latter typically present with hypotension due to vasodilatory shock and vasopressor refractoriness [53–57]. In addition, significantly increased systemic CXCL11 concentrations have recently been described in patients with hypertension [58], which could reflect an adaptive response to reduce vascular resistance. Our findings provide another example for the functional relevance of GPCR heteromers and insights into the regulation and biological functions of ACKR3 and AVPR1A, which could facilitate the development of improved pharmacological strategies to modulate vascular function.

## 3. Material and methods

### 3.1. Proteins, peptides and reagents

PE and aVP were purchased from Sigma-Aldrich, ubiquitin was from R&D Systems, CXCL11 and CXCL12 were from Protein Foundry, and TC14012 was from Tocris Biosciences. CXCL11_3–73_ was purified as N-terminal His_6_SUMO fusion proteins in *Escherichia coli* as previously described [7,59,60]. Cells were grown in Terrific Broth and induced with 1 mM isopropyl β-d-1-thiogalactopyranoside before being harvested and stored at −80°C. Cell pellets were lysed, and lysates were clarified by centrifugation (12 000*g* for 20 min). The supernatant and solubilized inclusion body pellets were loaded onto Ni-NTA resin and after 1 h proteins were eluted with 6 M guanidinium chloride, 50 mM Na_2_PO_4_ (pH 7.4), 300 mM NaCl, 500 mM imidazole, 0.2% sodium azide and 0.1% β-mercaptoethanol. The eluate was pooled and refolded via dilution overnight before cleavage of the His_6_SUMO fusion tag by Ulp1 protease for 4 h. The His_6_SUMO fusion tag and chemokine were separated using cation-exchange chromatography (SP Sepharose Fast Flow resin; GE Healthcare UK Ltd) and the eluate was subjected to reverse-phase high-performance liquid chromatography as a final purification. Proteins were frozen, lyophilized and stored at −20°C. Purification, folding and homogeneity of recombinant proteins were verified by SDS–PAGE, MALDI-TOF spectroscopy and ^1^H-^15^N HSQC NMR spectroscopy. The peptide analogues of transmembrane helix 2 (TM2; YILNLAIADLWVVLTIPVWVVDDD), TM4 (VVCILWLLAFCVSLPDTYYLDD) and TM7 (DDDLHVTQCLSLVHCCVNPVLYSFIN) of ACKR3 were as described previously [21].

### 3.2. Plasmids

The human AVPR1A cDNA sequence was from the AVPR1A-TANGO plasmid (Addgene, plasmid #66225). The hRLuc cDNA was PCR-amplified from pIRES-Myc-hCXCR4-Rluc, which was generously provided by Dr Michel Bouvier, with primers carrying the AgeI and XbaI sites at either end, respectively, and ligated with the C-terminus of AVPR1A at the corresponding sites. Human CXCR4 and ACKR3 cDNAs were from the CXCR4-TANGO (Addgene, plasmid #66262) and the ACKR3-TANGO (Addgene, plasmid #66265) plasmids. The EYFP cDNA was PCR-amplified from pEYFP with primers harbouring AgeI and XbaI sites and ligated with the C-terminus of ACKR3. All plasmids were confirmed by sequencing.

### 3.3. Cells

hVSMCs (primary aortic smooth muscle cells, ATCC PCS-100-012), A7r5 cells (ATCC-CRL-1444) and HEK293T cells (ATCC-CRL-11268) were purchased from American Type Culture Collection. hVSMCs were cultured in vascular basal media (ATCC PCS-100-030) supplemented with the vascular smooth muscle growth kit (ATCC PCS-100-042), containing 100 U ml^−1^ penicillin and 100 µg ml^−1^ streptomycin. hVSMCs were used between passages 2–5. HEK293T and A7r5 cells were cultured in high-glucose Dulbecco's Modified Eagle's Medium containing 10 mg ml^−1^ sodium pyruvate, 2 mM l-glutamine, 10% (vol/vol) FBS, 1× non-essential amino acids, 100 U ml^−1^ penicillin and 100 µg ml^−1^ streptomycin. The HTLA cell line, a HEK293 cell line stably expressing a tTA-dependent luciferase reporter and a β-arrestin 2–TEV fusion gene [27] were generously provided by the laboratory of Dr Bryan Roth and maintained in high-glucose Dulbecco's Modified Eagle's Medium supplemented with 10% (vol/vol) FBS, 1× non-essential amino acids, 100 U ml^−1^ penicillin, 100 µg ml^−1^ streptomycin, 50 µg ml^−1^ hygromycin B and 2 µg ml^−1^ puromycin. All cells were cultured in a humidified environment at 37°C, 5% CO_2_.

### 3.4. Proximity ligation assays

PLAs were performed as described in detail previously [19–21,61]. In brief, cells were grown and fixed on eight-well chamber slides (Nunc). Cells were fixed with 4% (wt/vol) paraformaldehyde for 15 min at room temperature and then blocked overnight at 4°C with 3% (wt/vol) BSA in PBS. To visualize individual proteins, slides were incubated with rabbit anti-HA (AbCam Ab9110), mouse anti-FLAG (Sigma-Aldrich F1804), rabbit anti-AVPR1A (Bioss BS-11598R), mouse anti-ACKR3 (R&D MAB42273), goat anti-CXCR4 (AbCam Ab1670) or mouse anti-phospho-MLC 2 (Ser19) (pMLC2, Cell Signaling Technology, 3675) at 37°C for 105 min in a humidifying chamber. To assess how permeabilization of the plasma membrane affects the PLA signals for pMLC2, cells were incubated in 0.5% Triton X-100 in PBS for 20 min at room temperature following fixation. To visualize receptor–receptor interactions, slides were incubated with a combination of rabbit anti-HA (AbCam Ab9110) and mouse anti-FLAG (Sigma F1804), rabbit anti-AVPR1A (Bioss BS-11598R) and mouse anti-ACKR3 (R&D MAB42273) or goat anti-CXCR4 (AbCam Ab1670) at 37°C for 105 min in a humidifying chamber. All antibodies were used in dilutions of 1 : 500. Slides were then washed with PBS and incubated (60 min at 37°C in a humidifying chamber) with secondary species-specific antibodies conjugated with plus and minus Duolink II PLA probes (1 : 5), as appropriate. Negative control slides were incubated with omission of one primary antibody. Slides were washed again with PLA wash buffer A (Duolink II) and then incubated with ligation-ligase solution (30 min at 37°C in a humidifying chamber), and also washed with PLA wash buffer A and then incubated with amplification polymerase solution (100 min at 37°C in a humidifying chamber). Slides were then washed twice with PLA wash buffer B (Duolink II), once with 0.01× PLA wash buffer B and allowed to dry. Slides were then mounted with a minimal volume of Duolink II mounting medium with 4′,6-diamidino-2-phenylindole dihydrochloride (DAPI) overnight, and PLA signals (Duolink In Situ Detection Reagent Red (*λ*_excitation/emission_ 598/634 nm) were identified as fluorescent spots under a fluorescence microscope (Carl Zeiss Axiovert 200M with EC Plan-Neofluor objective lenses (40 × /1.30 oil) equipped with Axio CamMRc5 (Carl Zeiss) and AxioVision Rel. 4.9.1 (Carl Zeiss) acquisition software) at room temperature. For each vision field 10 *z*-stack images in 1 µm sections were acquired and compressed. PLA signals were quantified using the Duolink Image Tool software (Sigma-Aldrich). Images were imported in merged.tiff formats containing both signal and nuclei channels. Merged images were visually verified for analytical quality. Comparisons and statistical analyses were performed only when PLA assays were performed on the same day in parallel experiments, and fluorescence microscopy was performed with the identical settings. For each experiment and condition, 10 randomly selected non-overlapping vision fields were analysed.

### 3.5. Deconvolution three-dimensional imaging

Deconvolution three-dimensional imaging was performed as described previously [19]. In brief, *z*-stack images were collected (from bottom to top, 20 sections of 0.5 µm) using identical acquisition parameters with a DeltaVision wide-field fluorescent microscope (Applied Precision, GE) equipped with a digital camera (CoolSNAP HQ; Photometrics), using a 1.4-numerical aperture 100× objective lens. Excitation light was generated using the Insight SSI solid-state illumination module (Applied Precision, GE), and images were deconvolved with the SoftWoRx deconvolution software (Applied Precision, GE). Following deconvolution, images were quantified by Imaris (Bitplane) software using the Surfaces feature function, generating surfaces around red puncta. Three-dimensional views of images were generated using the Surpass mode of Imaris software.

### 3.6. Co-immunoprecipitation analyses of receptor interactions

Co-immunoprecipitation experiments with hVSMC and HEK293T cells were performed using the ThermoScientific Pierce co-immunoprecipitation kit (cat. no. 26149), as described [21]. A total of 50 µg of rabbit anti-AVPR1A (Bioss BS-11598R), mouse anti-HA (Bioss bsm-50131M) or anti- rabbit IgG (AbCam Ab27478) were incubated with 50 µl of Amino Link Plus coupling resin for 180 min at room temperature. A cell lysate (1000 µg) was precleared with 50 µl of the control agarose resin slurry (60 min at 4°C). Immobilized anti-AVPR1A resin, anti-HA resin and anti-IgG resin were incubated with a precleared lysate for 48 h at 4°C. After incubation, the resins were washed three times with 200 µl of IP lysis/wash buffer, once with conditioning buffer, and protein was eluted using 60 µl of elution buffer. Samples were analysed by western blotting.

### 3.7. Western blotting

Western blotting with rabbit anti-AVPR1A (Bioss BS-11598R), rabbit anti-ACKR3 (AbCam Ab38089), goat anti-CXCR4 (Abcam Ab1670), rabbit anti-β_2_-AR (Abcam Ab36956), mouse anti-HA (Bioss bsm-50131M) or mouse anti-FLAG (Sigma-Aldrich F1804) in combination with anti-rabbit, anti-mouse (GE Healthcare) or anti-goat (Sigma-Aldrich) IgG horseradish peroxidase-linked whole antibody was performed as described previously [21].

### 3.8. Gene silencing via RNA interference

ACKR3 and CXCR4 siRNA gene silencing was performed as described previously [19,21,62]. In brief, cells were grown in 2 ml Accell siRNA delivery media per well (Dharmacon) in six-well plates (Nunc). Commercially available Accell ACKR3 and CXCR4 siRNA were reconstituted with 1× siRNA buffer to a stock concentration of 100 µM. Cells were then transfected with 1 µM ACKR3/CXCR4 siRNA and incubated for 72 h at 37°C, 5% CO_2_. Accell NT-siRNA pool was used as a negative control. After 72 h, cells were assayed for receptor cell surface expression and used for signalling experiments.

### 3.9. GPCR gene transfections

HEK293T cells were transiently transfected with 1.5 µg of DNA encoding either HA-AVPR1A, FLAG-ACKR3 or FLAG-CXCR4 with a combination of two GPCR encoding DNAs, as indicated, using Lipofectamine 3000 (ThermoScientific) as per the manufacturer's protocol. All cDNAs were from the Addgene Tango plasmids subcloned in pcDNA3 with either HA- or FLAG-tag at the N-terminus. Empty vector, pcDNA3, was used as a control. Twenty-four hours later, cells were fixed on chamber slides for PLA or lysed for co-immunoprecipitation experiments.

### 3.10. Inositol trisphosphate enzyme-linked immunosorbent assay

IP_3_ enzyme-linked immunosorbent assays were purchased from LS Bio and performed as per the manufacturer's protocol (LS BIO F10644). In brief, hVSMCs were grown to confluency in six-well dishes (Nunc) and then treated as described in the Results section. Cells were then washed once with cold PBS, 225 µl of cold PBS was added to each well and cells were lysed by ultrasonication. The cell lysate was centrifuged for 10 min at 4°C at 1500*g* to remove cellular debris. The total protein concentration in the supernatant was determined with the Bio-Rad DC Protein Assay as per the manufacturer's protocol (Bio-Rad 500-0116). Equivalent amounts of total protein were added to the ELISA strips diluted in the provided sample diluent (1 : 5 and 1 : 10). The assay was then completed as per the manufacturer's protocol. Optical densities were read on a Biotek Synergy II microplate reader (absorbance at 450 nm), and IP_3_ concentrations were extrapolated from the standard curve.

### 3.11. PRESTO-Tango β-arrestin recruitment assay

The PRESTO-Tango (parallel receptorome expression and screening via transcriptional output, with transcriptional activation following arrestin translocation) assay was performed as recently described [27]. The Tango plasmids were a gift from Dr Bryan Roth (all from Addgene). HTLA cells (2.5 × 10^5^ per well) were seeded in a six-well plate and transfected with 1.5 µg of the Tango plasmids using Lipofectamine 3000 (ThermoScientific). The following day, transfected HTLA cells (1 × 10^5^ cells per well) were plated onto poly-l-lysine precoated 96-well microplates and allowed to attach to the plate surface for at least 4 h prior to treatment. Proteins used for treatment were prepared in twice the final concentration in culture media, added at a 1 : 1 vol/vol ratio and incubated overnight at 37°C, 5% CO_2_ in a humidified environment. The following morning, media were removed from cell culture plates and replaced with a 100 µl 1 : 5 mixture of Bright-Glo (Promega) and 1× HBSS, 20 mM HEPES solution. Plates were then incubated at room temperature before measuring luminescence on a Biotek Synergy II plate reader.

### 3.12. Intermolecular bioluminescence resonance energy transfer assay

HEK293T cells were seeded in 12-well plates and transfected with AVPR1A-hRluc alone or together with plasmids encoding EYFP or ACKR3-EYFP using the Lipofectamine 3000 transfection reagent (ThermoScientific). For BRET titration assays, AVPR1A-hRluc at the fixed amount of 50 ng was co-transfected with increasing amounts of EYFP or ACKR3-EYFP. For BRET assays at a constant acceptor : donor ratio, increasing amounts of AVPR1A-hRluc and ACKR3-EYFP were co-transfected at a ratio of 1 : 10. In all assays, empty vector pcDNA3 was added to maintain the total cDNA amount for each transfection reaction constant. After an overnight incubation, cells were seeded in poly-l-lysine coated 96-well white plates and incubated again overnight. Cells were then washed with PBS and fluorescence was measured in a Biotek Synergy II plate reader (*λ*_excitation_ 485 nm, *λ*_emission_ 528 nm). For BRET measurements, coelenterazine H (Nanolight Technology) at 5 µM in PBS was added to the cells. After 10 min incubation at room temperature, luminescence was measured at 460 ± 40 and 528 ± 20 nm. The BRET signal is calculated as the ratio of RLU measured at 528 ± 20 nm over RLU at 460 ± 40 nm. The net BRET is calculated by subtracting the BRET signal detected when the AVPR1A-hRLuc was transfected alone. For titration experiments, net BRET ratios are expressed as a function of EYFP/total luminescence.

### 3.13. Receptor internalization assay

Assessment of receptor internalization upon agonist stimulation was achieved via flow cytometry. hVSMCs were treated with 1 µM of aVP or CXCL11 for various time points. The cells were washed once with ice cold DPBS, blocked and stained with rabbit anti-AVPR1A (Bioss BS-11598R) and mouse anti-ACKR3 (R&D MAB42273) antibodies at 1 : 200 dilution for 1 h on ice. Cells were then washed twice with FACS wash buffer (1× PBS, 2% FBS and 0.01% NaN_3_), and secondary antibodies were added at a 1 : 500 dilution and incubated for 30 min on ice (donkey anti-rabbit Alexa Fluor 647, Invitrogen A-31573 and donkey anti-mouse Alexa Fluor 488, Invitrogen A-21202). Cells were washed twice with FACS wash buffer and then fixed with 4% paraformaldehyde at room temperature for 15 min. After two additional washes, the cells were counted on a BD FACS Canto II (BD Biosciences) flow cytometer. The fluorescence intensities of at least 3 × 10^4^ cells were recorded and analysed using the FlowJo software (Tree Star).

### 3.14. Flow cytometry

Flow cytometry was used to assess equivalent recombinant Tango receptor expression. HTLA cells were labelled with rabbit anti-FLAG-Alexa Fluor 647 (R&D Systems IC8529R). The fluorescence intensities of at least 3 × 10^4^ cells were recorded and analysed using the FlowJo software (Tree Star).

### 3.15. Pressure myography

Pressure myography was performed as described in detail previously with slight modifications [7,63]. Male Sprague–Dawley rats (Harlan) were anaesthetized with 3.5% isoflurane. The mesentery was immediately removed and placed in 130 mM NaCl, 4.7 mM KCl, 1.18 mM KH_2_PO_4_, 1.17 mM MgSO_4_, 14.9 mM NaHCO_3_, 5.5 mM d-glucose, 0.026 mM EDTA and 1.16 mM CaCl_2_ aerated with 95% O_2_, 5% CO_2_ at 37°C. The animal was then euthanized by cardiectomy and bilateral decompression of the lungs. Third- or fourth-order mesenteric arteries were dissected free from the mesentery, mounted onto two glass cannulae with United States Pharmacopeia (USP) scale 11-0 sutures and pressurized to 80 mmHg in a DMT 110P pressure myograph system (DMT-USA). The intraluminal solution and the vessel bath solution were the same as described before. The vessel bath solution was continuously aerated with 95% O_2_, 5% CO_2_ throughout the experiment. The outer diameter (o.d.) of the pressurized vessel was then continuously measured and recorded via digital video-edge detection upon the addition of increasing doses of PE or aVP to the vessel bath.

### 3.16. Data analyses

Data are expressed as mean ± standard error of the mean from *n* independent experiments that were performed on different days. Data were analysed using GraphPad Prism v. 7 software. Unpaired Student's *t*-test or one-way analyses of variance (ANOVA) with Dunnett's multiple comparison post hoc test for multiple comparisons were used, as appropriate. Dose–response curves were analysed using nonlinear regression analyses. A two-tailed *p* < 0.05 was considered significant.

## Data accessibility

All plasmids are available from Addgene (http://www.addgene.org/). The original datasets generated and analysed during this study are available from the corresponding author on reasonable request.
